# Rock inhibitors in Alzheimer’s disease

**DOI:** 10.3389/fragi.2025.1547883

**Published:** 2025-03-20

**Authors:** Chao Zheng, Weiming Xia, Jianhua Zhang

**Affiliations:** ^1^ Azrieli Centre for Neuro-Radiochemistry, Brain Health Imaging Centre, Campbell Family Mental Health Research Institute, Centre for Addiction and Mental Health (CAMH), Toronto, ON, Canada; ^2^ Departments of Psychiatry, Chemistry, Pharmacology and Toxicology, University of Toronto, Toronto, ON, Canada; ^3^ Geriatric Research Education and Clinical Center, Bedford VA Healthcare System, Bedford, MA, United States; ^4^ Department of Pharmacology, Physiology and Biophysics, Boston University Chobanian and Avedisian School of Medicine, Boston, MA, United States; ^5^ Department of Biological Sciences, University of Massachusetts Kennedy College of Science, Lowell, MA, United States; ^6^ Department of Pathology, University of Alabama at Birmingham, Birmingham, AL, United States

**Keywords:** Alzheimer’s disease, fasudil, amyloid, phosphorylated tau, glucose metabolism, PET imaging, mitochondria, synapse

## Abstract

Alzheimer’s disease (AD) is the most common age-related neurodegenerative disease and cause of dementia. AD pathology primarily involves the formation of amyloid β (Aβ) plaques and neurofibrillary tangles containing hyperphosphorylated tau (p-tau). While Aβ targeted treatments have shown clinical promise, other aspects of AD pathology such as microgliosis, astrocytosis, synaptic loss, and hypometabolism may be viable targets for treatment. Among notable novel therapeutic approaches, the Ras homolog (Rho)-associated kinases (ROCKs) are being investigated as targets for AD treatment, based on the observations that ROCK1/2 levels are elevated in AD, and activation or inhibition of ROCKs changes dendritic/synaptic structures, protein aggregate accumulation, inflammation, and gliosis. This review will highlight key findings on the effects of ROCK inhibition in Aβ and ptau pathologies, as well as its effects on neuroinflammation, synaptic density, and potentially metabolism and bioenergetics.

## Introduction

Alzheimer’s disease (AD) is the leading cause of dementia, and in total, AD and other dementias affect 55 million people worldwide. AD diagnosis and staging rely on cognitive, functional, and behavioral tests, cerebrospinal fluid (CSF) and plasma analyses of amyloid β (Aβ) protein and hyperphosphorylated tau (ptau), as well as brain imaging analyses of amyloid and tau. Other biomarkers of AD pathogenesis include CSF or plasma Neurofilament light chain (NfL) and Glial Fibrillary Acidic Protein (GFAP) for the diagnosis of AD, MRI to assess brain atrophy, and positron emission tomography (PET) imaging of Fluorodeoxyglucose (FDG) uptake and synaptic vesicle protein 2A (SV2A) for assessing brain hypometabolism (Jack et al., 2024a; Hansson, 2024; Jack et al., 2024b) and loss of synaptic density (Wang et al., 2024a; Wang et al., 2024b; Mecca et al., 2022a; Mecca et al., 2022b; Mecca et al., 2020). It is well recognized that Aβ plaque accumulation often precedes cognitive symptoms by more than 10 years, with tau hyperphosphorylation, hypometabolism, and neuroinflammation also occurring throughout this clinically latent period. While coexisting pathologies such as alpha-synuclein or TDP43 accumulation may contribute to disease progression, neurofibrillary tangle formation and neurodegeneration generally occur closer to onset of cognitive symptoms. Nonetheless, a subpopulation of people preserve cognition in the presence of multiple pathologies (known as cognitive resilience), with unknown underlying mechanisms (Jack et al., 2024a).

Disease modifying therapies (DMT) for AD have been explored in the past several decades, mainly targeting Aβ, tau, neuroinflammation, enzymes that control neurotransmitter levels, and neurotransmitter receptors to alleviate clinical symptoms. Currently, only antibodies against Aβ have shown clinical promise in early-stage patients, however this treatment excludes those exhibiting amyloid-related imaging abnormality (ARIA) with micro hemorrhage (ARIA-H) and edema (ARIA-E), therefore limiting a broad application of this antibody-based Aβ therapy to patients with AD (Kato et al., 2016). Hence, ongoing studies and clinical trials are exploring tau, neuroinflammation, metabolism and bioenergetics as AD treatment targets.

Targeting Ras homolog (Rho)-associated kinase (ROCK) is among notable novel therapeutic approaches, based on the observation that ROCK activities are elevated in AD (Henderson et al., 2016; Weber and Herskowitz, 2021; Herskowitz et al., 2013), and ROCK activation leads to synaptic and metabolic dysfunction, inflammation, and gliosis (Weber and Herskowitz, 2021; Chong et al., 2017; Martin-Camara et al., 2021; Liu et al., 2007; Li et al., 2012; Jahani et al., 2018; Schinzari et al., 2012; Blazanin et al., 2024; Landry et al., 2020; Weber et al., 2021; Boros et al., 2019; Henderson et al., 2019; Swanger et al., 2015). Furthermore, preclinical studies with the ROCK inhibitor fasudil decreases Aβ and tau in 3xTg and Tau transgenic mice respectively, and lowers phosphorylated tau (ptau) and ameliorates cognitive deficits in APP/PS1 mice (Elliott et al., 2018; Guo et al., 2020a; Wei et al., 2021; Yan et al., 2021; Hamano et al., 2020; Tables 1, 2). Potential mechanisms driving these observations include regulation of AKT and autophagic pathways (Hamano et al., 2020; Preau et al., 2016; Liu H. et al., 2024; Yang et al., 2020; Gao et al., 2016; Gentry et al., 2016; Gurkar et al., 2013). This review will highlight the potential of ROCK inhibitors in the treatment of AD.

**TABLE 1:** Summary of ROCK inhibition in preclinical studies.

Area of interest	Inhibitor	Model	Observation	References
Protein aggregates	—	ROCK1 knockdown in mice primary neurons	Decreased Aβ40 levels in media after exposure to Aβ42	Henderson et al. (2016)
	Fasudil	AD neuro-spheroids	Decreased ptau	Giunti et al. (2023)
	H-1152 (ROCK2 specific)	Primary neurons, neuroblastoma cell lines and rTg4510 mice	Decreased ptau	Hamano et al. (2020)
	SR3677	5xFAD mice	Suppressed BACE1, decreased Aβ	Herskowitz et al. (2013)
	Fasudil	3xTg, PS19, and rTg4510 mice	Decreased Aβ and ptau	Elliott et al. (2018), Collu et al. (2024), Ouyang et al. (2024)
	Fasudil	APP/PS1 mice	Decreased Aβ and ptau	Guo et al. (2020a)
Behavior	Fasudil	rats with injected Aβ	Improved memory	Song et al. (2013)
	Fasudil	18 months-old rat	Improved memory	Huentelman et al. (2009)
		STZ treated rats	Improved memory	Hou et al. (2012), Kumar and Bansal (2018)
	Fasudil	APP/PS1 mice	Ameliorates cognitive deficits	Guo et al. (2020a)
Autophagy	Fasudil	AD neuro-spheroids	Increased AKT protein	Giunti et al. (2023)
	H-1152	Human neuroblastoma cells	Increased LC3II and lower p62, elevated proteasomal activities	Hamano et al. (2020)
	Y27632	Neuro2a cells	Activated proteosome and macroautophagy	Bauer et al. (2009)
	Fasudil	N9c2 cells	Elevates Beclin-1 and LC3II in high glucose	Gao et al. (2016)
	—	ROCK1 and 2 DKO in cardiomyocytes	Promotes starvation-induced autophagy, with increased LC3II, decreased AKT and mTOR	Shi et al. (2019)
	—	ROCK1 knockout in the heart	Decrease autophagy initiation p62 and LC3II in response to doxorubicin toxicity	Shi et al. (2018)
	—	ROCK1 knockout	Decrease autophagy in response to nutrient deprivation	Gurkar et al. (2013)
	—	ROCK2 knockout in cardiomyocytes	Compensatory ROCK1 overactivation and inhibition of autophagy	Shi et al. (2019)
	Fasudil	AAV delivered A53T alpha synuclein	Elevates LC3II/LC3I, Beclin1	Yang et al. (2020)
Mitophagy	SR3677	HEK293 cell line	Upregulation of Parkin-mediated mitophagy	Moskal et al. (2020)
Neuroinflammation	Fasudil	PS19 mice	Decreased GFAP	Ouyang et al. (2024)
	Fasudil	Rats with *i.c.v.* injected Aβ_1–42_	Decreased neuroinflammation	Song et al. (2013)
	Fasudil	STZ treated rats	Attenuated STZ effects TNFα, NFƙB, and eNOS	Kumar and Bansal (2018)
Synapse and dendrites	Fasudil	STZ treated rats	Attenuated synaptic changes	Hou et al. (2012)
	Fasudil	APP/PS1 mice	Increased dendritic arbors	Couch et al. (2010)
	Y27632	Hippocampal neurons	Increase in dendritic protrusion and density	Swanger et al. (2015)
Mitochondria	Y-27632	Cardiomyocytes	Attenuates RhoA effects on p-Drp1 and mitochondrial fragmentation	Brand et al. (2018)
	Ripasudil	Bovine corneal endothelia cells	Elevated oxidative phosphorylation genes, elevate oxygen consumption rates	Ho et al. (2022)
	Fasudil or Y27632	Senescent fibroblasts	Elevated coupling efficiency	Park et al. (2018)
	Fasudil or Y27632	2D cultured 3T3-L1 cells	attenuated mitochondrial respiration	Ida et al. (2022)
	GSK269962A	melanoma cells	suppresses mitochondrial respiration rate	Murali et al. (2024)
	Belumosudil	SMARCA4-mutant lung cancer cells	attenuated mitochondrial respiration	Schinzari et al. (2012)
	Knockdown of ROCK1 or Y27632	PC12 cells	attenuated MPP + induced mitochondrial fission and apoptosis	Zhang et al. (2019)
	Fasudil	PS19 mice	Decreased complex I–IV substrate linked oxygen consumption rate, decreased Complex I and II protein subunit levels	Ouyang et al. (2024)
	Fasudil	Primary neurons	attenuates NMDA-induced mitochondrial fragmentation	Martorell-Riera et al. (2015)
	Fasudil and Y27632	Primary neurons	Primary neurons	Gao et al. (2019)
Glycolysis	Fasudil	PS19 mice	Decreases tau transgene induced PKM1 levels	Ouyang et al. (2024)
	Fasudil	Drug network analyses	Alter gene expression in a similar fashion as 2-deoxy-glucose, a glycolysis inhibitor	Iorio et al. (2010)
	Y27632	Bovine corneal endothelia cells	Increased the contribution of glycolysis to ATP production	Ho et al. (2025)
	Fasudil or Y27632	2D cultured 3T3-L1 cells	attenuated glycolysis	Ida et al. (2022)
	GSK269962A	melanoma cells	suppresses glycolytic rate	Murali et al. (2024)
	Belumosudil	SMARCA4-mutant lung cancer cells	attenuated adaptive glycolysis enhancement in response to mitochondrial respiration	Blazanin et al. (2024)
Detrimental effects	—	ROCK1 knockout mice	Developmental defects	Julian and Olson (2014), Shimizu et al. (2005)
	—	ROCK2 knockout mice	Developmental defects	Julian and Olson (2014), Thumkeo et al. (2003)
	—	CaMKII-cre:ROCK2^f/f^ mice	Anxiety-like behavior	Greathouse et al. (2019)
	—	fasudil (oral gavage at 10 mg/kg for 30 days in 6 months old C57BL/6J, ROCK1^+/−^, or ROCK2^+/−^mice	Anxiety-like behavior	Greathouse et al. (2019)

**TABLE 2:** Summary of clinical and preclinical ROCK inhibitors.

Drug	Disease involved	Treatment type	Treatment effects	Status	Drug details	Molecular pathways involved	References
Fasudil (HA-1077)	Cerebral vasospasm, tauopathies (PSP-RS, CBS), ALS	Pan-ROCK inhibitor	Reduces vascular constriction; potential neuroprotective effects	Approved in Japan and China (cerebral vasospasm); In clinical trials for PSP-RS and CBS (US); Compassionate use for ALS; Not FDA-approved in the US	IC_50_: ∼740 nM (ROCK1/2); Half-life: 5.5 h; Oral: 20-80 mg TID; IV: 30 mg TID; Oral bioavailability: Not reported	RhoA/ROCK pathway	Hamano et al. (2020), Couch et al. (2010), Ono-Saito et al. (1999), Zhao et al. (2015), Guo et al. (2019)
Belumosudil (KD025)	Chronic graft-versus-host disease (cGvHD)	ROCK2-specific inhibitor	Immune modulation; reduces IL-21 and IL-17, Th17	FDA-approved (2021) for cGvHD; Oral dose: 200 mg QD	IC_50_: 100 nM (ROCK2), 3 µM (ROCK1); Tmax: 1.26-2.53 h; Half-life: 19 h; Oral bioavailability: 64%	STAT3 phosphorylation, IL-21, IL-17 in human CD4^+^ T cells	Lee et al. (2014), Zanin-Zhorov et al. (2014)
Netarsudil (AR-13324)	Open-angle glaucoma, ocular hypertension	Pan-ROCK inhibitor	Lowers intraocular pressure	FDA-approved (2017); Ophthalmic solution: 0.02% QD	IC_50_: 32 nM (ROCK1), 11 nM (ROCK2); Half-life: 175 min (human cornea), 16-17 h (rabbit aqueous humor); Oral bioavailability: Not applicable (topical)	ROCK pathway, norepinephrine transport inhibition	Dayal et al. (2019)
Ripasudil (K-115)	Glaucoma, ocular hypertension	Pan-ROCK inhibitor	Lowers intraocular pressure; Anti-inflammatory effects	Approved in Japan (2014); Ophthalmic solution: 0.4% BID	IC_50_: 51 nM (ROCK1), 19 nM (ROCK2); Half-life: 0.455 h; Clearance: 7 L/h; Oral bioavailability: Not applicable (topical)	ROCK pathway, anti-inflammatory pathways	Kaneko et al. (2016),/01; Wu et al. (2024)
SR3677	Alzheimer’s disease (AD)	ROCK2-specific inhibitor	Suppresses BACE1, reduces Aβ production	Not approved for human use	IC_50_: 3 nM (ROCK2), 56 nM (ROCK1); EC_50_: 57 nM (Parkin recruitment assay); Oral bioavailability: Not reported	BACE1 inhibition, Aβ reduction, Parkin recruitment	Herskowitz et al. (2013), Moskal et al. (2020), Feng et al. (2008)
BA-1049 (NRL-1049)	Cerebral cavernous malformations (CCM)	ROCK2-specific inhibitor	Reduces vascular pathology in CA mouse models	FDA-approved for Phase 1 investigational clinical trial for treating CCM	IC_50_: 0.59 µM (ROCK2), 26 µM (ROCK1); Dosage: 100 mg/kg/day or 10 mg/kg/day; Oral bioavailability: Not reported	ROCK activation, vascular pathology in CCM	McKerracher et al. (2020), Morrison et al. (2024)

## ROCK in Alzheimer’s disease-- Aβ, ptau, dendritic atrophy and neuroinflammation

ROCKs are serine/threonine kinases and downstream effectors of the Rho-GTPase RhoA. RhoA binding to GTP is facilitated by guanine nucleotide exchange factors (GEFs), and RhoA modulates ROCK activity in response to various signals and stresses. Further regulation by GTPase-activating proteins (GAPs) and guanine nucleotide dissociation inhibitors (GDIs) facilitates RhoA inactivation and inhibition (Julian and Olson, 2014; Aguilar et al., 2017). A recent structural study however, also presents the distinct possibility that ROCK2 activity can be constitutive, free of RhoA regulation (Truebestein et al., 2015). ROCK1 and 2, the two isoforms of ROCK, are on different chromosomes in humans. ROCK1 is expressed ubiquitously, and ROCK2 is highly expressed in specific tissues including the brain, heart and skeletal muscle. ROCK isoforms have distinct intracellular locations and effector molecules, variably regulating the cytoskeleton and dendritic morphology (Henderson et al., 2019; Newell-Litwa et al., 2015). ROCK1 and ROCK2 knockout mice exhibit strain-dependent developmental abnormalities and low survival rate, while those survived were largely normal. Mice which survive whole body and tissue specific knockout exhibit protective phenotypes in cardiac hypertrophy, diabetic kidney disease, vascular remodeling and atherosclerosis (Julian and Olson, 2014). C57BL/6J, ROCK1^+/−^, or ROCK2^+/−^ mice treated by the ROCK inhibitor fasudil, and ROCK2 forebrain excitatory neuron conditional knockout mice under CaMKII-cre, exhibit anxiety-like behavior (Table 1; Weber et al., 2021; Thumkeo et al., 2003; Shimizu et al., 2005; Zhang et al., 2006; Greathouse et al., 2019). These studies suggest that ROCK1 and 2 play an essential role in development, yet demonstrate detrimental effects in certain diseases (Julian and Olson, 2014). Furthermore, ROCK inhibition can be potentially beneficial while long term use may result in unwanted side effects (Weber et al., 2021; Thumkeo et al., 2003; Shimizu et al., 2005; Zhang et al., 2006; Greathouse et al., 2019).

Extensive evidence points to the potential role of ROCK in AD. In *postmortem* AD brains, it has been demonstrated that ROCK1 and ROCK2 protein levels are elevated compared to controls (Henderson et al., 2016; Herskowitz et al., 2013). Primary neurons exposed to Aβ42 oligomers exhibited elevated pLIMK1, a downstream target of ROCK (Henderson et al., 2016). Furthermore, ROCK1 heterozygous mice exhibited decreased Aβ40 levels in the brain, and knockdown of ROCK1 in primary neurons decreased Aβ40 levels in media after exposure to Aβ42, highlighting a potential pathological mechanism by which ROCK1 exacerbates Aβ accumulation (Henderson et al., 2016). In SH-SY5Y cells however, ROCK1 knockdown increased and ROCK2 knockdown decreased secreted Aβ40, suggesting cell type and culture conditions may impact Aβ processing (Herskowitz et al., 2013). ROCK inhibitor SR3677 treatment in 5xFAD mice decreased Aβ40 and 42 and suppressed BACE1 activity (Herskowitz et al., 2013). Furthermore, ROCK inhibitor fasudil decreased ptau in AD neuro-spheroids (Giunti et al., 2023), and ROCK2 selective inhibitor H-1152 decreased ptau in primary neurons and neuroblastoma cell lines (Hamano et al., 2020). Furthermore, ROCK inhibitor Y-27632 increased dendritic protrusion and density in hippocampal neurons (Swanger et al., 2015; Table 1).

ROCK inhibition activates degradation pathways which remove protein aggregates, as evidenced in the following examples. ROCK inhibitor fasudil elevated AKT1 protein levels in AD neuro-spheroids (Giunti et al., 2023). ROCK2 inhibitor H-1152 elevated LC3II and lowered p62 that are consistent with elevation of autophagy, as well as elevated chymotrypsin-like 20S and trypsin-like 26S proteasomal activities (Hamano et al., 2020). Y27632 also decreased multiple protein aggreates in Neuro2a cells, and the beneficial effects are partially mediated by autophagy and attenuated by proteasome and autophagy inhibitors (Bauer et al., 2009). Both ROCK1 and ROCK2 knockdown exhibited similar outcomes as Y27632 treatment (Bauer et al., 2009). The observation that Y27632 further decreases protein aggregates when combined with ROCK1 and 2 double knockdown is intriguing, suggesting either Y27632 has additional targets than ROCK1/2, or the knockdowns are incomplete (Bauer et al., 2009).

ROCK inhibition mediates protein degradation through various pathways. It has been shown that ROCK and mTOR activities are highly correlated, and ROCK2 knockdown decreased mTOR, an autophagy regulator which downregulates autophagy upon phosphorylation (Gentry et al., 2016). Furthermore, ROCK1 and ROCK2 double knockout in cardiomyocytes promotes starvation-induced autophagy, with increased LC3II and decreased AKT, mTOR, and ULK signaling (Shi et al., 2019). Other autophagy mediators also respond to ROCK inhibition in a stress and cell type specific manner. In H92c cells exposed to high glucose, ROCK inhibitor fasudil increased Beclin1, LC3II/LC3I ratio, and Bcl-2 levels, decreased ROCK1/2 levels. In addition, high glucose-induced apoptosis in these cells is attenuated by fasudil, and fasudil’s anti-apoptotic effect is abolished by autophagy inhibitor 3-MA (Gao et al., 2016). In mice with AAV delivered A53T alpha-synuclein, fasudil treatment elevated LC3II/LC3I ratio, Beclin1 and p-Bcl2/Bcl2 in the brain (Yang et al., 2020).

Effects of ROCK1 or 2 disruption on autophagy are stress and cell type dependent. Disruption of ROCK1 in the heart is cardioprotective against doxorubicin toxicity, associated with decreased accumulation of p62 and LC3II, likely due to decreased autophagy initiation which is mediated by Beclin1 phosphorylation (Shi et al., 2018). In HeLa and 293T cells, ROCK1 interacts with Beclin1 upon starvation (Gurkar et al., 2013). In HeLa and EJ cells, ROCK1 mediates starvation-induced autophagy through phosphorylation of Beclin1, and this is attenuated by ROCK inhibitor Y-27632. Embryonic fibroblasts, EJ cells, and heart tissue from ROCK1 whole body knockout exhibited decreased autophagy following nutrient deprivation (Gurkar et al., 2013). ROCK2 knockout in cardiomyocytes led to compensatory ROCK1 overactivation, associated with inhibition of autophagy, and increased cardiac fibrosis (Shi et al., 2019). This latter work in cardiomyocyte specific ROCK knockout mice further highlights potential developmental and compensatory roles of ROCK, at least in certain tissues, when either ROCKs is disrupted.

Related to autophagy and with potentials to affect mitochondrial function, a recent compound screening study shows that ROCK inhibitors upregulate Parkin-mediated mitophagy (Moskal et al., 2020). The effects of ROCK inhibitors on autophagy and mitophagy flux have largely been studied *in vitro*. Further investigation is needed to understand whether and how autophagy and mitophagy are responsible at least in part for the effects of ROCK inhibition on Aβ, ptau, synaptic, and neuroinflammatory phenotypes *in vivo*.

Many *in vivo* studies have explored the effects of ROCK inhibition on neuroinflammation, cognition, and synaptic phenotypes. In rats, ROCK inhibitor fasudil decreased neuroinflammation induced by *i.c.v.* injected Aβ_1–42_, and improved memory, concurrent with attenuation of neuronal loss and NF-κB activation (Song et al., 2013). In 18 month-old rats, fasudil improved learning and memory in water radial-arm and water maze tests (Huentelman et al., 2009). In streptozotocin (STZ) injected rats, fasudil reversed STZ effects on learning and memory, synaptophysin levels, synaptic structural alterations, and levels of p-LIMK2 and p-cofilin (Hou et al., 2012). PI3K inhibitor wortmannin and NOS blocker L-NAME inhibited the effects of Fasudil in attenuating STZ induced changes in memory AChE, TNFα, NFƙB, and eNOS (Kumar and Bansal, 2018).

In 3xTg mice, fasudil treatment decreased Aβ (Elliott et al., 2018). In APP/PS1 mice fasudil decreased Aβ, ptau, and apoptosis; attenuated synaptophysin/Gap43 loss; and improved cognition (Guo et al., 2020a). Tau transgenic mice (rTg4510) treated with fasudil exhibit lower ptau (Hamano et al., 2020). Furthermore, fasudil in APP/PS1 mice increased dendrite arbors (Couch et al., 2010), and in 3xTg mice significantly changed transcriptomes in the rostral cortices with genes downregulated in AD, Parkinson’s disease and Huntington’s disease upregulated by fasudil, including ATP6V1E1 and ATP6V0B which are involved in lysosomal function (Killick et al., 2023). We have shown that fasudil significantly decreased ptau and GFAP in the hippocampus of PS19 mice (Collu et al., 2024; Ouyang et al., 2024). The effects of fasudil on neuroinflammation may be indirectly mediated by ptau or involve regulation of homeostasis of pro-inflammatory and anti-inflammatory astrocytes or microglia (Guo et al., 2020b; Zhang et al., 2013; Ding et al., 2009; Zhao et al., 2023; Chen et al., 2017).

## ROCK inhibitors in human use and clinical trials

Because of the cell and animal studies on ROCK inhibitors, several promising ROCK inhibiting compounds have been considered for human use and clinical trials (Table 2). Fasudil is the most frequently used ROCK inhibitor in cell and animal models, despite being a weak ROCK inhibitor (*IC*_*50*_ of ∼740 nM for ROCK1 and 2) (Ono-Saito et al., 1999). Fasudil is approved and used for the treatment of cerebral vasospasm in Japan and China (Hamano et al., 2020; Couch et al., 2010; Ono-Saito et al., 1999; Zhao et al., 2015; Guo et al., 2019), and is currently in clinical trials for tauopathies of Progressive Supranuclear Palsy-Richardson Syndrome and Corticobasal Syndrome in the US (NCT04734379). Fasudil is also under compassionate use for amyotrophic lateral sclerosis (ALS) in the US (NCT03792490) and Europe (2017-003676-31). Clinical trials for oral formulation range from 20 to 80 mg 3 times a day or *i.v.* 30 mg 3 times a day, with a half-life of 5.5 h (PubChem as of 12 December 2024).

Belumosudil (2-[3-[4-(1H-Indazol-5-ylamino)-2-quinazolinyl]phenoxy]-N-(1-methylethyl) acetamide, also known as KD025) is a ROCK2-specific inhibitor with an IC_50_ = 100 nM (IC_50_ = 3 µM for ROCK1, PubChem) (Lee et al., 2014; Zanin-Zhorov et al., 2014), and received FDA approval for chronic graft *versus* host disease (cGvHD) in 2021. ROCK2 knockdown or KD025 treatment decreases STAT3 phosphorylation, production of IL-21 and IL-17 in human CD4^+^ T cells, and shifts the balance between Th17 and regulatory T cells (Zanin-Zhorov et al., 2014), and is thus considered an immune modulator. Oral administration results in 64% bioavailability and Tmax at 1.26–2.53 h, with mean elimination half-life of 19 h (Belumosudil | C26H24N6O2 | CID 11950170 - PubChem, as of 12 December 2024).

Netarsudil ([4-[(2S)-3-amino-1-(isoquinolin-6-ylamino)-1-oxopropan-2-yl]phenyl] methyl2,4-dimethylbenzoate, also known as AR-13324) has IC_50_ at 32 nM for ROCK1 and 11 nM for ROCK2 (Dayal et al., 2019), while also inhibits norepinephrine transport. Netarsudil was proven effective in lowering intraocular pressure. The US FDA approved netarsudil for clinical use in 2017 as ophthalmic solution at 0.02% for open-angle glaucoma or ocular hypertension. *In vitro* incubation of human corneal tissue with netarsudil identified a half-life of 175 min (PubChem as of 12 December 2024), and 16–17 h from aqueous humor in rabbits (Netarsudil Mesylate Monograph for Professionals - Drugs.com as of 12 December 2024).

Ripasudil (4-Fluoro-5-[[(2S)-hexahydro-2-methyl-1H-1,4-diazepin-1-yl] sulfonyl] isoquinoline, also known as K-115)) is another pan-ROCK inhibitor (with the IC_50_ at 51 and 19 nM^68^, respectively for ROCK1 and 2) approved as one drop of 0.4% ophthalmic solution twice daily for the treatment of glaucoma and ocular hypertension in 2014 in Japan. It is cleared by the kidney at 7 L/h and biological half-life is 0.455 h (Ripasudil|C15H18FN3O2S|CID 9863672 - PubChem, as of 12 December 2024). Short-term (8 weeks) and long-term (24 months) studies have also observed anti-inflammatory effects in human patients (Wu et al., 2024).

SR3677 [IC_50_ of 3 nM for ROCK2 and 56 nM for ROCK1 (Feng et al., 2008)] also suppresses β-site APP cleaving enzyme 1 (BACE1) activity, and attenuates Aβ production in an AD mouse brain (Herskowitz et al., 2013). SR3677 EC_50_ is 57 nM in an assay for Parkin recruitment to damaged mitochondria (Moskal et al., 2020). Considering that ROCK1 and ROCK2 knockdown had opposite effects on endogenous human Aβ levels in SH-SY5Y cells, and that inhibition by SR3677 decreases both endogenous Aβ in SH-SY5Y cells, and Aβ in 5XFAD mouse brains, ROCK2 specific inhibitors such as SR3677 may be of importance in AD treatment. However, this drug has not been approved for human use, and is thus so far not a prime drug repurposing candidate.

NRL-1049 (synonyms: BA-1049) (1-(1-isoquinolin-5-ylsulfonylpiperidin-4-yl)ethanamine; dihydrochloride, IC_50_ = 0.59 µM and 26 µM for ROCK2 and ROCK1, MedChemExpress website and US20170313680A1, accessed 14 December 2024) exhibits benefits in Ccm1+/−Msh2−/− and Ccm3+/-Trp53−/− cavernous angioma (CA) mouse models which show vascular pathology with ROCK activation (McKerracher et al., 2020). The treatment was initiated from weaning with daily 100 mg/kg/d (Ccm1+/-Msh2−/−) or 10 mg/kg/d (Ccm3+/−Trp53−/−) until 105 and 77 days of age. The underlying mechanisms are not fully understood, and this compound is currently being evaluated in a Phase 1 investigational clinical trial for treating CCM in patients (Morrison et al., 2024).

Additional ROCK inhibitors exist (Ye et al., 2024; Koch et al., 2018; Mani et al., 2022; Feng et al., 2016), and a subset can be found in MedChemExpress (which listed 112 inhibitors and two activators, and comparisons of specificity to ROCK1 or 2 including IC_50_ when available, as of 15 December 2024) or PubChem.

## ROCK inhibition, metabolism, and *in vivo* outcome measurements

As discussed above, potential mechanisms of ROCK inhibitors include the potential to upregulate Parkin-mediated mitophagy, which can in turn impact mitochondrial function (Moskal et al., 2020). In a transcription drug effect network analysis, the effects of fasudil on gene expression and levels of LC3II are similar to 2-deoxy-glucose in their regulation of autophagy genes (Iorio et al., 2010). 2-Deoxy-glucose has been shown to target hexokinase, the first enzyme in glycolysis, thus impacting glucose metabolism and the pentose phosphate pathway (Dodson et al., 2013a; Dodson et al., 2022; Benavides et al., 2013; Dodson et al., 2013b). We have shown that *in vivo* delivery of high fasudil doses in PS19 mice can decrease glycolytic enzyme Pkm1 in broad regions of the brain, and decrease mitochondrial complex IV subunit I in striatum and thalamic regions (Ouyang et al., 2024).

Effects of ROCK inhibition on oxidative stress and metabolism have been shown in multiple tissues. Fasudil administration alongside insulin treatment improved blood flow in patients with obesity-related metabolic syndrome with insulin resistance. In the presence of vitamin C, fasudil no longer exerted additional benefit in blood flow, consistent with a role of oxidative stress in the vascular dysfunction in these patients (Schinzari et al., 2012). ROCK1 is increased in human fatty liver diseases (Schinzari et al., 2012), and overexpression of ROCK1 in the liver promotes adiposity, insulin resistance and lipid accumulation in mice with a high-fat diet (Schinzari et al., 2012). Knockout ROCK1 in the liver attenuated steatosis and hyperglycemia in obese diabetic (ob/ob) mice (Schinzari et al., 2012). Furthermore, the diabetic drug metformin inhibits ROCK1 activity (Schinzari et al., 2012). ROCK inhibition by H1152 or ROCK2 knockdown promotes the generation of insulin-expressing pancreatic beta-like cells from hPSCs (Ghazizadeh et al., 2017). Effects of ROCK inhibition on mitochondria have been shown in cardiomyocytes, where activation of RhoA increases phosphorylation of Drp1 and formation of smaller mitochondria, and ROCK inhibition by Y27632 attenuates RhoA effects on Drp1 phosphorylation and mitochondrial fission (Brand et al., 2018).

Effects of ROCK inhibition on mitochondrial bioenergetics have also been directly measured in corneal endothelial cells (Ho et al., 2022). In these cells, ROCK inhibitor ripasudil upregulated genes for oxidative phosphorylation; concurrent with an increase in basal, maximal, proton leak, ATP production linked, and reserve capacity oxygen consumption rate; while glycolytic rate remained unchanged (Ho et al., 2022). The changes in oxygen consumption rate are associated with increased phosphorylation of AMPK, and are attenuated by AMPK inhibitor dorsomorphin (Ho et al., 2022). Inhibition of mitochondrial function by oligomycin attenuated effects of ripasudil on cell migration (Ho et al., 2022). ROCK inhibitor Y27632 treatment has been shown to enhance hexokinase HK2 localization to the mitochondria in the presence of monensin which facilitates sodium transport across the cell membrane and increases cellular energy demand. This is associated with increased contribution of glycolysis to ATP production (Ho et al., 2025). In senescent fibroblasts, fasudil or Y27632 elevated coupling efficiency based on ATP linked oxygen consumption rate normalized to basal oxygen consumption rate (Park et al., 2018).

Effects of ROCK inhibition on mitochondria dynamics can differ by cell type. In 2D cultured 3T3-L1 cells, ripasudil and Y27632 both attenuated mitochondrial respiration and glycolytic function following suppression by BIM-A^87^. In SMARCA4-mutant lung cancer cells, ROCK inhibition by KD025 (Belumosudil) suppresses both mitochondrial respiration and adaptive increase in glycolysis induced by inhibition of oxidative phosphorylation (Blazanin et al., 2024). In melanoma cells that had elevated glycolysis, ROCK inhibition by GSK269962A suppressed glycolytic enhancement, oxygen consumption rate, as well as cell proliferation (Murali et al., 2024). ROCK inhibitor Y27632 has also been shown to promote mitochondrial transfer via tunneling nanotubes in retinal pigment epithelium. However, mitochondrial respiration was not enhanced by Y27632 indicating that beneficial effects from ROCK inhibition were mediated by promotion of mitochondrial transfer, not regulation of mitochondrial respiration (Yuan et al., 2024).

In the brain, studies of the effects of ROCK on metabolism have focused on the hypothalamus, where ROCK1 levels are upregulated by leptin, and decreased by a high-fat diet. Overexpression of ROCK1 in the hypothalamus decreases food intake and body weight, and fasudil treatment in prenatal and postnatal rats increases body weight (Huang et al., 2013). In primary neurons, fasudil treatment alleviated the decrease in mitochondrial membrane potential induced by Aβ (Gao et al., 2019). Fasudil and Y27632 have also been shown to attenuate NMDA-induced mitochondrial fragmentation (Martorell-Riera et al., 2015). Knockdown of ROCK1 or Y27632 treatment in PC12 cells attenuated MPP+ induced mitochondrial fission and apoptosis, and Y27632 treatment *in vivo* attenuated MPTP-induced motor symptoms (Zhang et al., 2019). These studies with MPP+ and MPTP highlight the potential of ROCK inhibition in treating Parkinson’s disease, as MPTP has been shown to induce *parkinsonism* in humans and mitochondrial dysfunction is suggested to contribute to Parkinson’s disease (Cannon and Greenamyre, 2010; Crabtree and Zhang, 2012).

We have recently reported that high doses of fasudil in PS19 mice resulted in significantly decreased ptau, GFAP, mitochondrial complex I and II protein subunits, and complex I–IV substrate linked oxygen consumption rate in the cortex (Ouyang et al., 2024). Beyond our own study, the effects of ROCK inhibition on mitochondrial electron transport chain function and glycolysis have not been critically investigated in cortical and hippocampal neurons or in the context of AD *in vivo*. Considering the accumulating evidence demonstrating insufficient mitophagy and mitochondrial bioenergetics in AD, and other neurodegenerative disease, and that ROCK inhibition can affect mitophagy and mitochondrial function in multiple cells, tissues and stress conditions (Moskal et al., 2020; Mani et al., 2022; Chen et al., 2020; Wang et al., 2020; Cai and Jeong, 2020; Redmann et al., 2016; Jeong et al., 2022; John and Reddy, 2021; Weidling and Swerdlow, 2020; Fang et al., 2019; Jia et al., 2024; Carling et al., 2024; Udeochu et al., 2023; Swerdlow, 2023; Liu Y. et al., 2024; Tai et al., 2023; Trushina et al., 2022; Apolloni et al., 2022; Luo et al., 2021; Cunnane et al., 2020), ROCK inhibition may also impact pathogenesis-associated bioenergetic dysfunction in AD and other neurodegenerative diseases.

Metabolic dysregulation is a significant feature of AD (Author Anonymous, 2020; Austad et al., 2021), and decreased glucose metabolism occurs in AD brains (Holscher, 2011; Szablewski, 2017; Goedert and Spillantini, 2006). Among well-established brain imaging procedures for diagnosis of AD and other dementias in standard clinical work-up (Chételat et al., 2020), [^18^F]FDG (Fluorodeoxyglucose) PET readily demonstrates local cerebral metabolic rate of glucose consumption (Kato et al., 2016). Glycolysis and mitochondrial energy production are intimately linked. Mitochondrial DNA mutations, deficiencies in electron transport chain activities, and deficits in glucose and lipid metabolism in AD support the link between mitochondrial and metabolic dysfunction in AD (Fang et al., 2019; Swerdlow et al., 2017; Swerdlow et al., 2014; Butterfield and Halliwell, 2019; Kunkle et al., 2019; Bloom and Norambuena, 2018; Holper et al., 2019; Swerdlow et al., 2010). The “mitochondrial cascade hypothesis” is based on evidence that mitochondrial dysfunction can exacerbate AD related pathologies (Weidling and Swerdlow, 2020; Holper et al., 2019; Swerdlow and Khan, 2004; Swerdlow, 2020). Despite many studies *in vitro* and in other disease models, the mechanisms linking ROCK inhibition and metabolic regulation in AD, and the pathways connecting glycolysis, mitochondrial dysfunction, and AD pathology are still largely unclear, primarily due to a lack of tools for detection of these metabolic activities *in vivo* in distinct cell types and brain regions. The development and application of multiple *in vivo* imaging tools are essential to further understand and dissect this mechanistic link.

There were demonstrated relationships between FDG PET, brain volumes based on volumetric magnetic resonance imaging (MRI), and tau PET in disease cases and brain regions (Matthews et al., 2024). Hypometabolism has been shown to have high predictive value for cognitive impairment, and/or decline rate (Matthews et al., 2024; Drzezga et al., 2005; Iaccarino et al., 2019; Ou et al., 2019; Koops et al., 2024). Furthermore, distinct patterns of FDG PET have been found for different mixed pathologies (Minoshima et al., 2022). Additionally, a recent study demonstrated more extensive loss of proteins involved in mitochondrial dynamics and bioenergetics in rapidly progressive AD compared to typical AD, while ptau accumulation more closely resembles non-AD control than typical AD, consistent with how ptau accumulation is slower, or that rapid progressive AD and typical AD have different etiologies (Xiyang et al., 2024). Hypometabolic mismatch has also been observed in mixed AD with Lewy body disease (LBD), in that patients with amyloid and alpha-synuclein pathologies exhibit worse hypometabolism relative to tau pathology and brain atrophy (Duong et al., 2024). These new observations further support the importance of developing therapeutics to protect against metabolic and bioenergetic deficits in the brain.

A number of studies have used the concept of targeting bioenergetic and mitochondrial mechanisms as a therapeutic strategy for age-related neurodegenerative diseases (Weidling and Swerdlow, 2020; Fang et al., 2019; Cunnane et al., 2020; Swerdlow et al., 2010; Hill et al., 2019; Rigotto and Basso, 2019). Thus far, a small percentage of AD drug development pipelines target metabolism and bioenergetics (e.g., Two compounds: metformin and tricaprilin, a ketone body precursor compound, in phase III) (Cummings et al., 2024; Cummings et al., 2023). Drugs that target metabolism must be critically evaluated for their potential use in treating AD, either alone or in combination with other therapeutic strategies. Recent clinical trials, evoke and evoke+, are randomized, double-blind, placebo-controlled phase 3 trials investigating the efficacy, safety, and tolerability of once-daily oral semaglutide in early-stage symptomatic AD (Cummings et al., 2025), and this glucagon-like peptide-1 receptor agonist is already approved for the treatment of type 2 diabetes and obesity (Fessel, 2024). While the outcomes of the trial will be available in the near future, it is anticipated to achieve 30% improvement of AD, as benefit to cognitive function in neurodegenerative disorders is expected following treatment (Fessel, 2024; Fan et al., 2020). Interestingly, there have been studies linking GLP-1 and the ROCK signaling pathway in the peripheral tissues (Fan et al., 2020; Kong et al., 2014). Both GLP-1 and Y27632 decrease glucotoxicity-induced increase of stress fibers, and GLP-1 inhibited glucotoxicity-induced activation of RhoA/ROCK (Kong et al., 2014). Furthermore, GLP-1 agonist liraglutide has been shown to ameliorate myocardial hypertrophy in hypertensive rats, potentially through ROCK2 decrease (Fan et al., 2020), as in H9C2 cells, GLP-1 decreased ROCK2, and Y27632 further decreased AngII effects on hypertrophic gene ANP expression when combined with GLP-1 (Fan et al., 2020).

Current FDA approved AD treatments are antibodies which decrease cerebral Aβ, including aducanumab, lecanemab and donanemab, and both lecanemab and donanemab have shown clinical benefits in clinical trials (Gandy, 2023). These advances provide hope for effective AD treatment, while also further revealing challenges and opportunities. Antibody treatment has serious side effects including the occurrence of amyloid related imaging abnormality (ARIA) with micro hemorrhage and edema (brain swelling or bleeding), and might not be as effective for patients with greater tau pathology (Gueorguieva et al., 2023a; Sims et al., 2023; Reardon, 2023; Manly and Deters, 2023; Gueorguieva et al., 2023b).

Most clinical trials have not used the FDA-approved FDG PET as an endpoint measurement. [^18^F]FDG is a radioactive glucose analog widely used in clinical and research settings worldwide for PET imaging to assess metabolic activity and detect abnormalities in tissues, particularly for cancer, cardiac, and neurological conditions. Mechanistically, fluorodeoxyglucose crosses the blood-brain barrier and is phosphorylated by hexokinase to fluorodeoxy-6-phosphate, acting as a competitive substrate with glucose at the first step of glycolysis. Because of the deoxy substitution, it cannot be further metabolized through the glycolysis pathway, and thus remains in the tissue to be detected through imaging.

**FIGURE 1:**
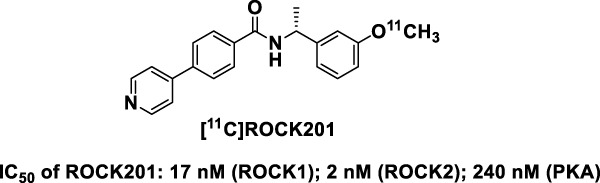
Structure of the first-in-class ROCK ligand [11C]ROCK201 for imaging studies. IC50 for ROCK1, ROCK2 and PKA are indicated, demonstrating higher affinity to ROCKs.

PET imaging with suitable radioligands can quantify the distribution of proteins of interest through the measurement of radioactivity distribution in living subjects. Such radioligands may reveal, non-invasively, differences in quantity and distribution between healthy and diseased states. To determine *in vivo* effects of ROCK inhibition, we have developed the first-in-class ROCK ligand [^11^C]ROCK201 for imaging studies to determine the target occupancy (Figure 1; Zheng et al., 2022). We have evaluated the imaging characteristics of this brain permeable ROCK2 radiotracer in rodent and nonhuman primate brains, and demonstrated fast brain kinetics and specific binding signals. This development will facilitate the study of ROCK, the effects of ROCK inhibition on Aβ, ptau, and metabolism, how these pathologies act together in AD progression, and whether ROCK inhibition attenuates AD.

## Conclusion, urgent needs, challenges, and future directions

**FIGURE 2:**
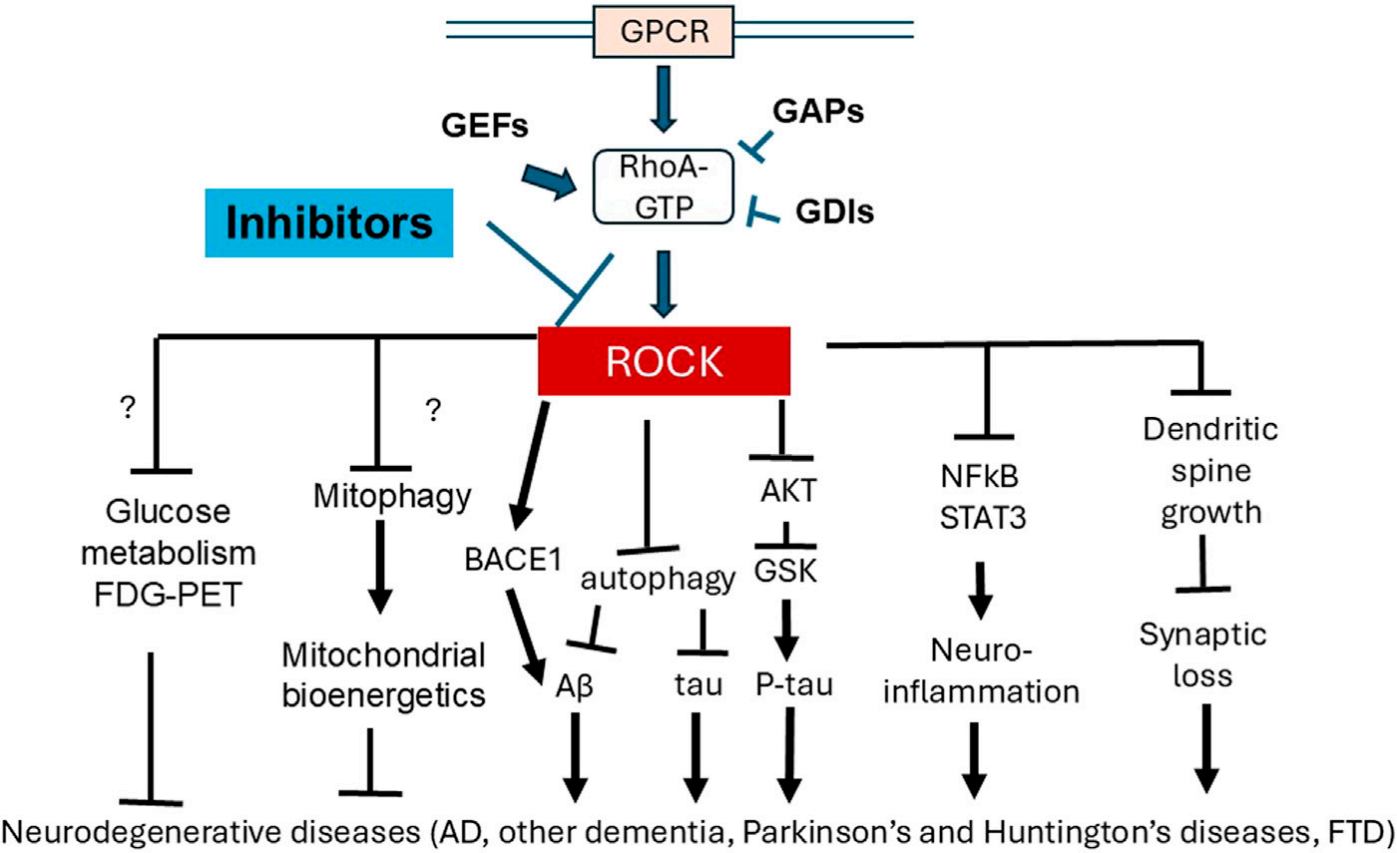
Inhibition of ROCK have multiple downstream functions which can provide benefit for neurodegenerative diseases. ROCK are downstream effectors of the Rho-GTPase RhoA, which binds to GTP, and regulated by guanine nucleotide exchange factors (GEFs), GTPase-activating proteins (GAPs) and guanine nucleotide dissociation inhibitors (GDIs). Because of a converging role of ROCK in regulating multiple downstream functions, ROCK inhibitors have been developed, which have demonstrated extensive pre-clinical ability to ameliorate AD pathology, including Aβ and ptau (potentially through BACE, autophagy and AKT regulation), neuroinflammation (potentially through NFkB and STAT3 regulation) and synaptic loss (potentially through cytoskeleton regulation and dendritic spine growth). ROCK inhibition in non-neuronal tissue and disease models has also shown effects on metabolism including glycolysis and mitochondrial bioenergetics, but effects of ROCK inhibition on neuronal metabolism remains to be explored. FDG-PET can aid future studies to elucidate these potential effects of ROCK inhibition on metabolism.

Metabolic and bioenergetic deficits are significant features of AD, thus intervention strategies for AD ideally not only address Aβ and ptau pathology but also protect against synaptic loss, neuroinflammation, metabolic and bioenergetic deficiencies. Studies addressing this objective are still in urgent need. ROCK inhibitors have shown effects on autophagy, mitophagy, synapse and neuroinflammation, in addition to well-established results concerning pathogenic Aβ and ptau. Furthermore, some ROCK inhibitors have been approved for human use in treating other diseases, and thus have the potential to serve as effective drugs for AD treatment. Overall, ROCK inhibitors have a broad range of effects, including as immune modulators (graft vs. host disease), in attenuating Aβ production, in recruiting Parkin to damaged mitochondria, and in the treatment of intra-ocular pressure. ROCK inhibitors target multiple downstream signaling pathways, including but not limited to: cytoskeleton regulation (e.g., myosin light chain phosphatase and cofilin); NFkB and STAT3 pathways; the expression of angiotensin-converting enzyme, eNOS, IL21 and 17; autophagy pathway proteins (mTOR, Beclin1, Bcl2, LC3II, p62, ATP6V1E1 and ATP6V0B); HK2 and Parkin targeting to the mitochondria; Drp1 phosphorylation, and levels of the glycolytic enzyme PKM1 (Figure 2).

Major barriers in developing ROCK inhibitor-based AD therapies are as follows:

1. Research on AD therapies remain predominately focused on Aβ and ptau, which are AD specific biomarkers, while neuroinflammation, synaptic loss, metabolism and mitochondrial function are implicated in many diseases and conditions beyond AD. These pathologies not particularly specific to AD prevent commitment of effort and resources to study their contribution to disease pathogenesis and their potential as drug targets. AD has broad pathological phenotypes and it remains a possibility that cognitive resilience is dependent on phenotypes and regulation beyond Aβ and ptau. Thus, investigating AD pathogenesis processes and regulation needs to be broader than simply on Aβ and ptau.
2. Determining metabolic and mitochondrial bioenergetic changes in a cell type specific manner, *in vivo*, with single cell resolution is still technically challenging. Super resolution non-invasive imaging of mitochondrial membrane potential, ATP/ADP ratio, or complex activities is still lacking. Detecting changes in these parameters *in vivo* may enhance our conceptual understanding of cognitive neuroscience and neurodegenerative disease by facilitating the measurement of real-time activities while cognitive tests are being performed, and longitudinally during disease progression.
3. ROCK1 and 2 knockout mice exhibit developmental abnormalities and excitatory neuron specific ROCK2 knockout mice exhibit anxiety behavior. ROCK2 knockout has been shown to lead to overactivation of ROCK1 and inhibition of autophagy. Some evidence also suggests that current ROCK inhibitors may have off-target effect as represented by the observation that Y-27632 further decreases protein aggregates when ROCK1 and 2 are both knocked down. Similarities and differences among ROCK inhibitors concerning brain penetration, off-target effects, on-target side effects, systemic effects to peripheral tissues, dose, frequency, and duration of treatment, and effectiveness in subtypes and substages of AD, need to be further evaluated in both AD animal models and human AD iPSC/neuro-spheroids to define the beneficial window of treatment. As has been said, “the dose makes the poison.”
4. ROCK1/2 target multiple substrates and enzymes, and inhibiting ROCK may have broad side effects. ROCK is involved in various signaling pathways that control cell migration, proliferation, and apoptosis. Systemic inhibition of ROCK may have unexpected consequences that impair bodily functions, such as vascular regulation and immune responses. The multi-target properties of ROCK inhibition make it challenging to control the dose and duration of treatment to modulate specific pathways. Yet, multi-target drugs may be what we need to treat a multi-faceted disease such as AD. Indeed, there are ample examples of multi-target drugs including but not limited to GLP-1 agonists for treating diabetes.

With the advance of artificial intelligence and the ability to process big data, we expect to see great strides in this field. As some of the ROCK inhibitors are in human clinical trials or approved to be used for illnesses other than AD, analyses of data available in human populations will also provide crucial information for accelerating the refinement of therapies for AD treatment. We hope that some of the studies will soon benefit AD patients.
